# Low Expression of Slit2 and Robo1 is Associated with Poor Prognosis and Brain-specific Metastasis of Breast Cancer Patients

**DOI:** 10.1038/srep14430

**Published:** 2015-09-24

**Authors:** Fengxia Qin, Huikun Zhang, Li Ma, Xiaoli Liu, Kun Dai, Wenliang Li, Feng Gu, Li Fu, Yongjie Ma

**Affiliations:** 1 Department of Breast Cancer Pathology and Research Laboratory, Key Laboratory of Breast Cancer Prevention and Therapy (Ministry of Education); 2 Department of Neuro-oncology and Neurosurgery, Key Laboratory of Cancer Prevention and Therapy of Tianjin, Tianjin Medical University Cancer Institute and Hospital, Tianjin, China; 3 Department of tumor cell biology, Key Laboratory of Cancer Prevention and Therapy of Tianjin; Tianjin Medical University Cancer Institute and Hospital, National Clinical Research Center for Cancer, Huanhu West Road, Hexi District, Tianjin, PR China, 300060

## Abstract

Brain metastasis is a significant unmet clinical problem in breast cancer treatment. It is always associated with poor prognosis and high morbidity. Recently, Slit2/Robo1 pathway has been demonstrated to be involved in the progression of breast carcinoma. However, until present, there are no convincing reports that suggest whether the Slit2/Robo1 axis has any role in brain metastasis of breast cancer. In this study, we investigated the correlation between Slit2/Robo1 signaling and breast cancer brain metastasis for the first time. Our results demonstrated that (1) Invasive ductal carcinoma patients with low expression of Slit2 or Robo1 exhibited worse prognosis and brain-specific metastasis, but not liver, bone or lung. (2) Lower expression of Slit2 and Robo1 were observed in patients with brain metastasis, especially in their brain metastasis tumors, compared with patients without brain metastasis. (3) The interval from diagnosis of breast cancer to brain metastasis and brain metastasis to death were both much shorter in patients with low expression of Slit2 or Robo1 compared with the high expression group. Overall, our findings indicated that Slit2/Robo1 axis possibly be regarded as a significant clinical parameter for predicting brain metastasis in breast cancer patients.

Brain metastasis of breast cancer is a severe clinical problem that strongly affects patients’ quality of life. Recently, the incidence of brain metastasis in breast cancer patients is increasing due to improved methods of detection; an estimated 10% to 30% of all breast cancer patients will eventually develop brain metastasis^1^,^2^. Although most patients received multimodality treatment, once brain metastasis occurred, the 1- and 2-year survival rates were only about 20% and 2%, respectively^3^. Therefore identifying the genetic and epigenetic events leading to development of brain metastasis and designing novel diagnostic and therapeutic procedures are clinically significant.

Slit2 and Robo1 were first identified in the development of central nervous system^4^,^5^. Subsequently, various studies have shown that their promoters are hypermethylated in several different types of cancers and Slit2/Robo1 axis participates in different cellular processes, such as proliferation and migration^6^,^7^,^8^,^9^. Knocking down of Slit2 expression in gastric cancer cells promoted cells motility^10^. Slit2 overexpressing breast cancer cells displayed the reduced tumor growth and Slit2 or Robo1-deficient mammary epithelium led to hyperplasia after xenografts transplantation^11^. Although Slit2/Robo1 is linked to aberrant growth and migration of tumor epithelial cells, which consequently resulted in metastatic spread of cancer cells, the clinical significance of this axis in brain metastasis of breast cancer is unknown.

In this clinicopathologic study, we found that breast cancer patients with low expression of Slit2 or Robo1 exhibited brain-specific metastasis, but not liver, bone or lung. The expression of Slit2 and Robo1 in their paired brain metastasis was both much lower than primary tumors. Furthermore, the mean interval (from diagnosis of breast cancer to brain metastasis) and mean survival (from diagnosis of brain metastasis to death) were both significantly shorter in patients with low expression of Slit2 or Robo1 than the high expression group. Taken together, our studies demonstrate a novel role for Slit2/Robo1 axis in brain metastasis of breast cancer and probably provide a new therapeutic option in patients with brain metastasis.

## Results

### Expression of Slit2 and Robo1 in breast benign lesions, DCIS and IDC tissues

**Figure 1:**
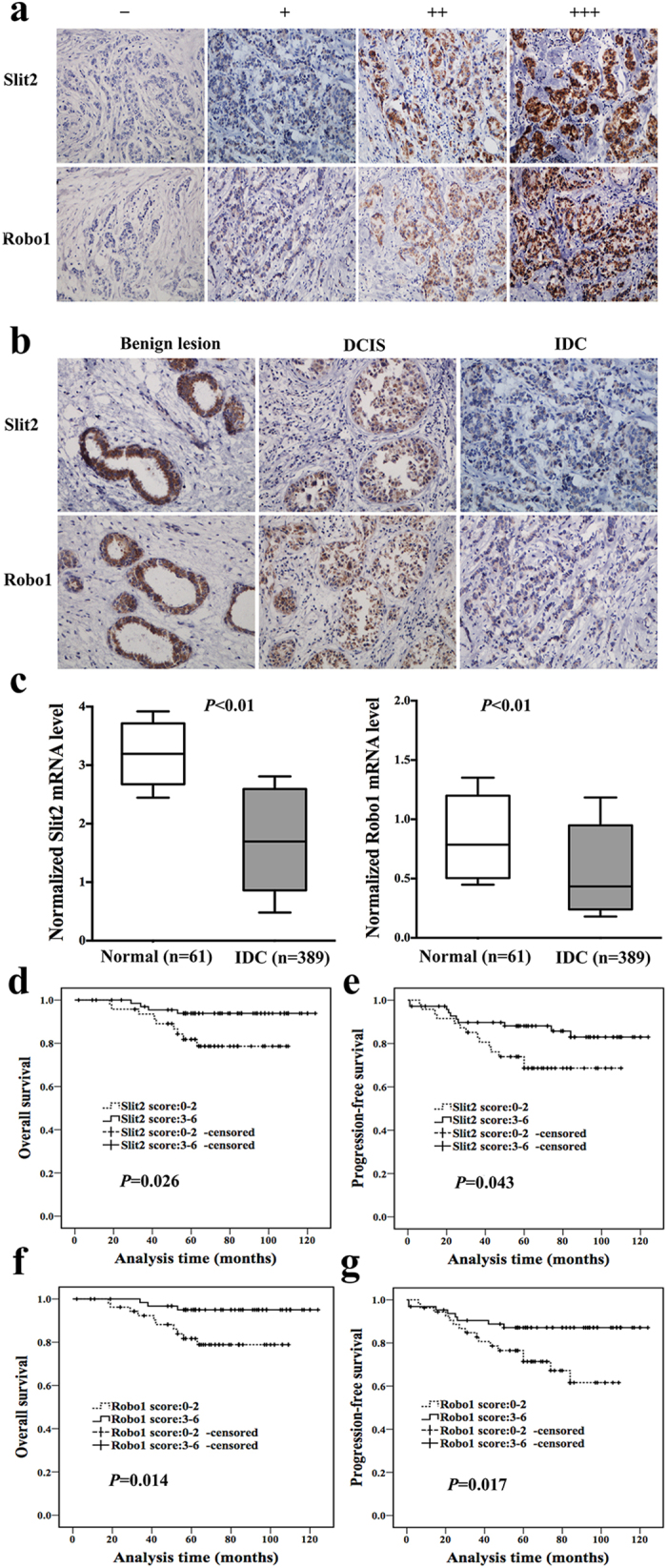
Expression of Slit2 and Robo1 were both down-regulated in breast cancer tissues and correlated with worse prognosis of breast cancer patients. (**a**) Varying degree staining intensity of Slit2 and Robo1 protein in invasive ductal carcinoma specimens: (−) no staining; (+) definite but weak staining; (++) moderate staining; and (+++) strong staining. (**b**) Immunohistochemical staining of Slit2 (upper part) and Robo1 (lower part) in clinical specimens of benign breast lesions, ductal carcinoma *in situ* (DCIS) and invasive ductal carcinoma (IDC). Photographs were taken at a magnification of 200×. (**c**) Normalized mRNA levels of Slit2 and Robo1 which were analyzed by gene expression profiling data from ONCOMINE (www.oncomine.org) Database. The data included 61 normal breast tissue samples and 389 IDC tissue samples. (**d**) Overall survival (OS) curves of IDC patients with Slit2 expression. (*P* = 0.026; log-rank test). (**e**) Progression-free survival (PFS) curves of IDC patients with Slit2 expression (*P* = 0.043). (**f**) OS curves of IDC patients with Robo1 expression (*P* = 0.014). (**g**) PFS curves of IDC patients with Robo1 expression (*P* = 0.017).

A total of 196 specimens from patients including 118 with invasive ductal carcinoma (IDC), 44 with ductal carcinoma *in situ* (DCIS) and 34 with benign breast lesions were analyzed by immunohistochemistry. The immunohistochemical staining of Slit2 and Robo1 was assessed and the intensity of staining was shown in representative images of Fig. 1a. In breast tissues, Slit2 and Robo1 were mainly located in the cytoplasm of epithelial cells of the mammary gland ducts. None stromal cells showed immunoreactivity for Slit2 or Robo1. Additionally, we found the expression of Slit2 and Robo1 were both gradually decreased from benign breast lesions to DCIS, and to IDC (Fig. 1b). 14.7% (5/34) of benign lesions, 36.4% (16/44) of DCIS and 39.8% (47/118) of IDC tissue specimens showed low expression of Slit2 (*P* = 0.024, Table 1). DCIS and IDC specimens showed a statistically reduced expression of Slit2 compared to that of benign lesions (*P* = 0.032, *P* = 0.007), while no statistical difference of Slit2 expression was found between DCIS and IDC (*P* = 0.687, Table 1). A similar expression pattern was observed for Robo1 in the benign lesions, DCIS and IDC tissue specimens (*P* = 0.029, Table 1). The negative controls of immunohistochemical staining of Slit2 and Robo1 were shown in the supplementary Fig. S1.

Next, we analyzed mRNA expression levels of Slit2 and Robo1 in gene expression profiling data sets from breast cancer and normal tissues. These data were mined from the publicly available ONCOMINE (www.oncomine.org) database, including breast cancer tissues (n = 389) and normal breast tissues (n = 61). The validation data confirmed that mRNA expression levels of Slit2 and Robo1 were down-regulated in breast cancer tissues compared with their corresponding normal tissues (*P* < 0.01, Fig. 1c).

Slit2 mediates multiple functions by binding to its Robo1 receptor, and then we investigated the relationship between Slit2 and Robo1 expression in the 118 IDC patients. In this study, we found the protein expression of Slit2 was positively correlated with Robo1 (*r*_s_ = 0.538, *P* = 0.000, Supplementary Table S1).

### Low expression of Slit2 or Robo1 in IDC patients indicated worse prognosis

To explore the prognostic significance of Slit2/Robo1 axis in breast cancer patients, we analyzed 118 IDC patients with complete clinical follow-up. The OS (overall survival) of patients with low Slit2 expression was much shorter than that of patients with high Slit2 expression (*P* = 0.026, Fig. 1d), and low Slit2 expression was also associated with a decreased PFS (progression-free survival) (*P* = 0.043, Fig. 1e). The median overall survival was 66.8 (range, 18–110) months in patients with low Slit2 expression and 75.6 (range, 2–124) months in patients with high Slit2 expression, respectively. The median PFS was 60.3 (range, 6–110) months and 71.5 (range, 1–124) months in low or high Slit2 expression patients, respectively.

In the Kaplan-Meier analysis, patients with low Robo1 expression also exhibited a significantly shorter OS and DFS. The mean overall survival was 64.7 months in patients with low Robo1 expression and 78.3 months in high Robo1 expression patients (Fig. 1f, *P* = 0.014). The mean PFS of patients with low or high Robo1 expression was 58.4 and 74.3 month, respectively (Fig. 1g, *P* = 0.017).

To further assess the independent prognostic value of Slit2 and Robo1, we carried out subset analyses according to other possible prognostic factors using Cox regression analysis. Univariate analysis indicated that Slit2 expression, Robo1 expression, pathological grade, lymph node metastasis and cTNM were significantly associated with shortened OS (Table 2). In multivariate Cox regression analysis, Slit2 and Robo1 were both independent prognostic factors for OS, when stratified by tumor size, pathological stage, lymph node metastasis, and cTNM (Table 2).

### Low expression of Slit2 or Robo1 positively associated with brain metastasis of breast cancer

In the present study, we found a specific positive correlation between low expression of Slit2 (*P* = 0.036) or Robo1 (*P* = 0.014) and brain metastasis in 118 IDC patients (Table 3). No significant associations were identified between Slit2 or Robo1 expression and distant metastasis to other organs, such as bone, lung and liver (Table 3).

**Figure 2:**
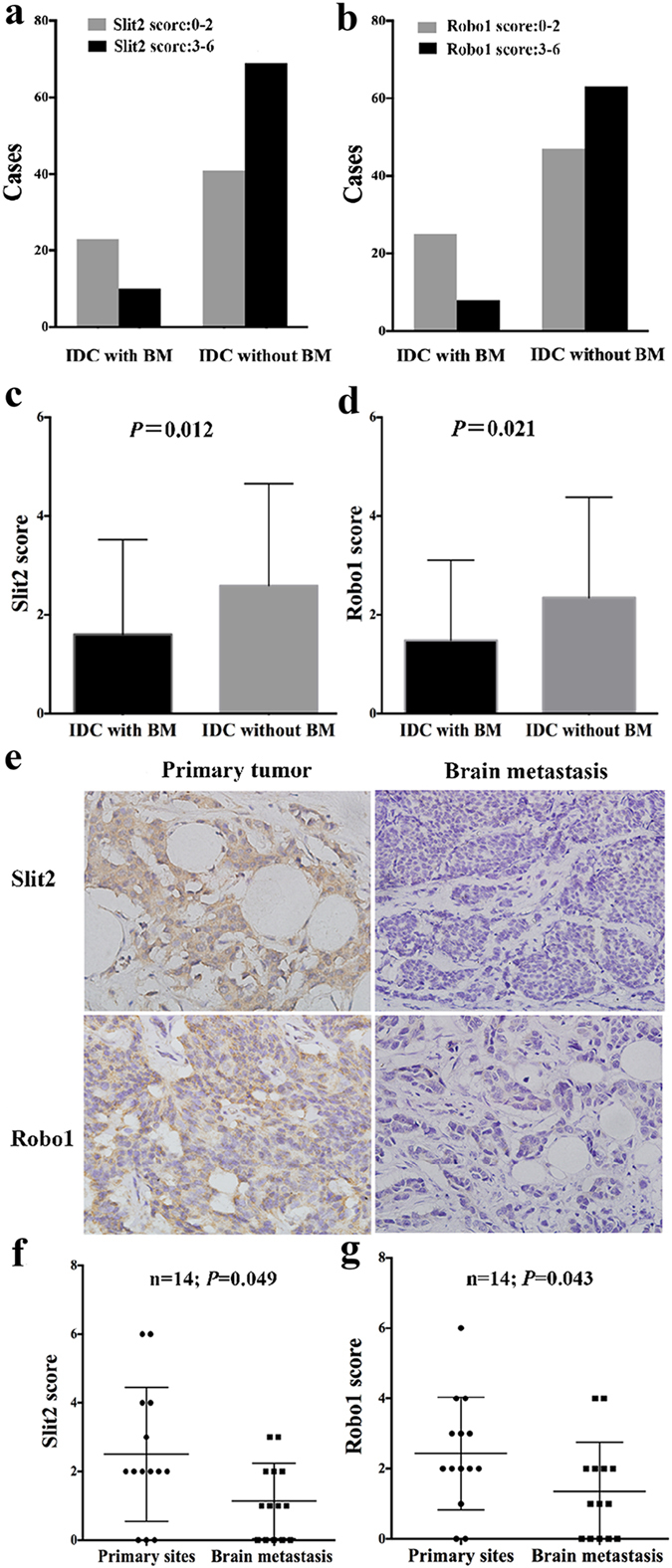
Patients with brain metastasis (BM) have lower expression of Slit2 and Robo1 than that without BM, and furthermore both Slit2 and Robo1 are down-regulated in brain metastasis specimens compared with their primary tumor sites. (**a**) In the group of patients with BM, most patients (23/33) showed low expression of Slit2, while in the group of without BM, most patients (69/110) exhibited high expression of Slit2. (**b**) In the group of patients with BM, most patients (25/33) patients showed low expression of Robo1, while most patients (63/110) exhibited high Robo1 expression in the group of without BM. (**c**) The average Slit2 IHC score in patients with BM (mean ± SEM: 1.606 ± 0.334) was much lower than patients without BM (mean ± SEM: 2.591 ± 0.197) (Mann-Whitney U test, *P* = 0.012). Columns are presented as mean with SD. (**d**) The average Robo1 IHC score in patients with BM (mean ± SEM: 1.485 ± 0.282) was much lower than that in patients without BM (mean ± SEM: 2.345 ± 0.194) (Mann-Whitney U test, *P* = 0.021). Columns were presented as mean with SD. (**e**) Representative immunohistochemical images of Slit2 and Robo1 expression in brain metastasis specimens and their primary tumor sites. Photographs were taken at a magnification of 400×. (**f**) Comparison of Slit2 IHC score in brain metastasis specimens and their primary tumor sites (n = 14). (**g**) Comparison of Robo1 IHC score in brain metastasis specimens and their primary tumor sites (n = 14). Wilcoxon signed ranks test was used and data was presented as mean ± SD.

The relationship between Slit2 or Robo1 expression and breast cancer brain metastasis was further investigated in an enlarged cohort of 33 IDC patients with brain metastasis and 110 IDC patients without brain metastasis. Compared with the patients without brain metastasis, Slit2 and Robo1 expression were both lower significantly in patients with brain metastasis. In patients with brain metastasis, 23/33 (69.7%) patients exhibited low Slit2 expression, while in the patients without brain metastasis, 41/110 (37.3%) patients showed low expression of Slit2 (*P* = 0.001, Fig. 2a, Table 4). For Robo1, 25/33 (75.8%) patients with brain metastasis showed low Robo1 expression, and 47/110 (42.7%) patients without brain metastasis exhibited low Robo1 expression (*P* = 0.001, Fig. 2b, Table 4). It was noting that there was no significant difference between the two groups in age, tumor size, pathological stage, and lymph node metastasis status, suggesting the specific important role of Slit2 and Robo1 in breast cancer brain metastasis (Supplementary Table S2).

Moreover, the average score of Slit2 and Robo1 in patients with brain metastasis was significantly lower than that in patients without brain metastasis. The average IHC score of Slit2 in patients with or without brain metastasis was 1.606 ± 0.334 and 2.591 ± 0.197, respectively (Z = −2.519, *P* = 0.012, Fig. 2c). The average IHC score of Robo1 in patients with brain metastasis was 1.485 ± 0.282; while the average IHC score of Robo1 was 2.345 ± 0.194 in patients without brain metastasis, (Z = −2.311, *P* = 0.021, Fig. 2d).

Furthermore, we compared the expression level of Slit2 and Robo1 in the paired 14 primary sites of IDC and their corresponding brain metastasis sites. Representative images for Slit2 and Robo1 were shown in Fig. 2e. We found that compared with primary sites, the brain metastasis specimens showed a significant lower expression of both Slit2 (Z = −1.971, *P* = 0.049) and Robo1 (Z = −2.207, *P* = 0.043). The average IHC score of Slit2 was 2.500 ± 0.522 and 1.143 ± 0.294 in primary and brain metastasis sites, respectively (Fig. 2f). Meanwhile, the IHC score of Robo1 was 2.429 ± 0.429 (primary tumors) and 1.357 ± 0.372 (brain metastasis specimens), respectively (Fig. 2g).

### Low expression of Slit2 or Robo1 in patients with brain metastasis indicated worse prognosis

**Figure 3:**
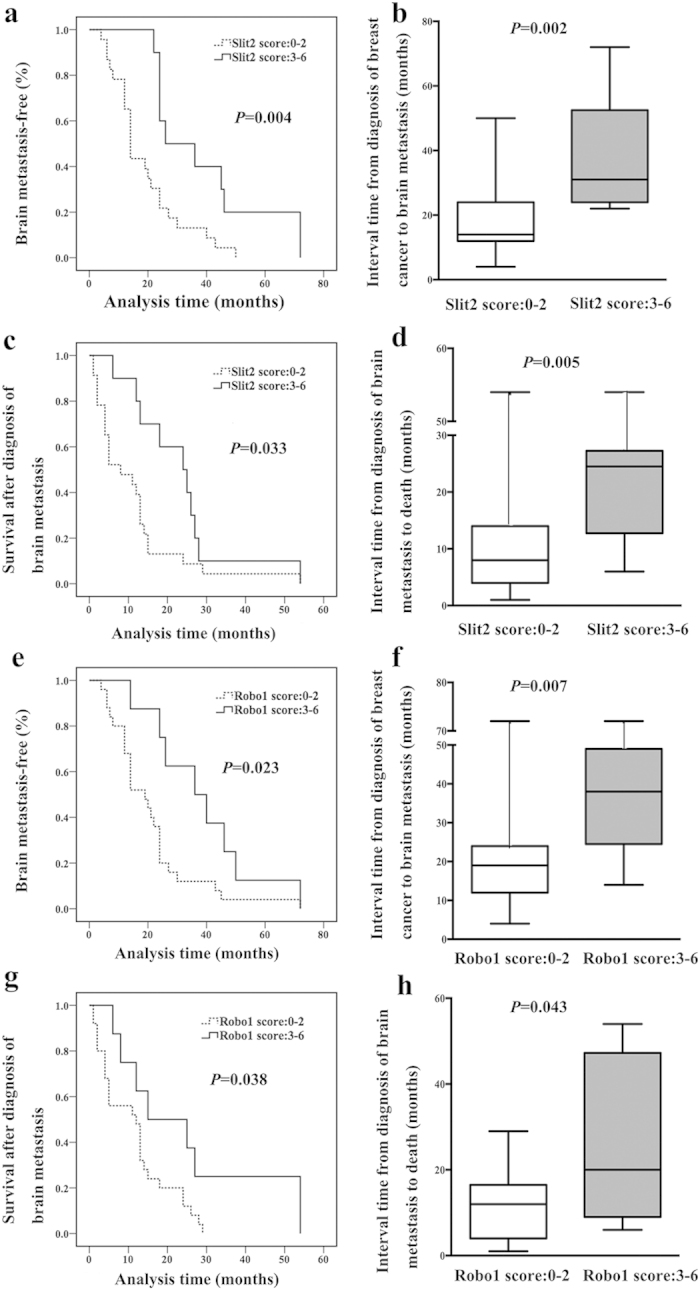
Patients with low expression of Slit2 or Robo1 exhibited earlier occurrence of brain metastasis and shorter survival after diagnosis of brain metastasis. (**a**) Brain metastasis occurred earlier in patients with low Slit2 expression than high Slit2 expression group (log-rank test, *P* = 0.004). (**b**) The median interval from diagnosis of breast cancer to brain metastasis in patients with low expression of Slit2 was much shorter than the high Slit2 expression group (Mann-Whitney U test, *P* = 0.002). (**c**) The survival after diagnosis of brain metastasis was worse in low Slit2 expression patients than high Robo1 expression group (log-rank test, *P* = 0.033). (**d**) The median interval from brain metastasis to death of patients with low expression of Slit2 was significantly shorter than that of high Robo1 expression patients (Mann-Whitney U test, *P* = 0.005). (**e**) Brain metastasis occurred earlier in patients with low Robo1 expression than high Robo1 expression group (log-rank test, *P* = 0.023). (**f**) The median interval from diagnosis of breast cancer to brain metastasis in patients with low expression of Robo1 was much shorter than high Robo1 expression group (Mann-Whitney U test, *P* = 0.007). (**g**) The survival after diagnosis of brain metastasis was worse in low Robo1 expression patients than high Robo1 expression group (log-rank test, *P* = 0.038). (**h**) The median interval from brain metastasis to death of patients with low expression of Robo1 was significantly shorter than high Robo1 expression patients (Mann-Whitney U test, *P* = 0.043).

Next, we analyzed the interval from diagnosis of breast cancer to brain metastasis and from detection of brain metastasis to death respectively in the 33 patients with brain metastasis. Brain metastasis occurred earlier in patients with low Slit2 expression than high Slit2 expression group (*P* = 0.004, Fig. 3a). The median interval from diagnosis of breast cancer to brain metastasis in patients with low expression of Slit2 (14 months) was much shorter than the high Slit2-expression group (31 months, Z = −3.146, *P* = 0.002, Fig. 3b). Prognosis of the 33 patients with brain metastases was evaluated by Kaplan-Meier analysis. The survival after diagnosis of brain metastasis was shorter in low Slit2 expression patients than that of high Slit2 expression group (*P* = 0.033, Fig. 3c). The median interval from brain metastasis to death of patients with low expression of Slit2 (8 months) was significantly shorter than that of high Slit2 expression patients (24 months, Z = −2.827, *P* = 0.005, Fig. 3d).

We also found that brain metastasis occurred earlier in patients with low Robo1 expression than the high Robo1 expression group (*P* = 0.023, Fig. 3e). The median interval from diagnosis of breast cancer to brain metastasis in patients with low expression of Robo1 (19 months) was much shorter than the high Robo1 expression group (38 months, Z = −2.720, *P* = 0.007, Fig. 3f). The survival after diagnosis of brain metastasis was worse in low Robo1 expression patients than that of high Robo1 expression group (*P* = 0.038, Fig. 3g). The median interval from brain metastasis to death of patients with low expression of Robo1 (12 months) was significantly shorter than that of high Robo1 expression patients (20 months, Z = −2.021, *P* = 0.043, Fig. 3h). Overall, patients with low expression of Slit2 or Robo1 exhibited earlier occurrence of brain metastasis and shorter survival after diagnosis of brain metastasis.

**Figure 4:**
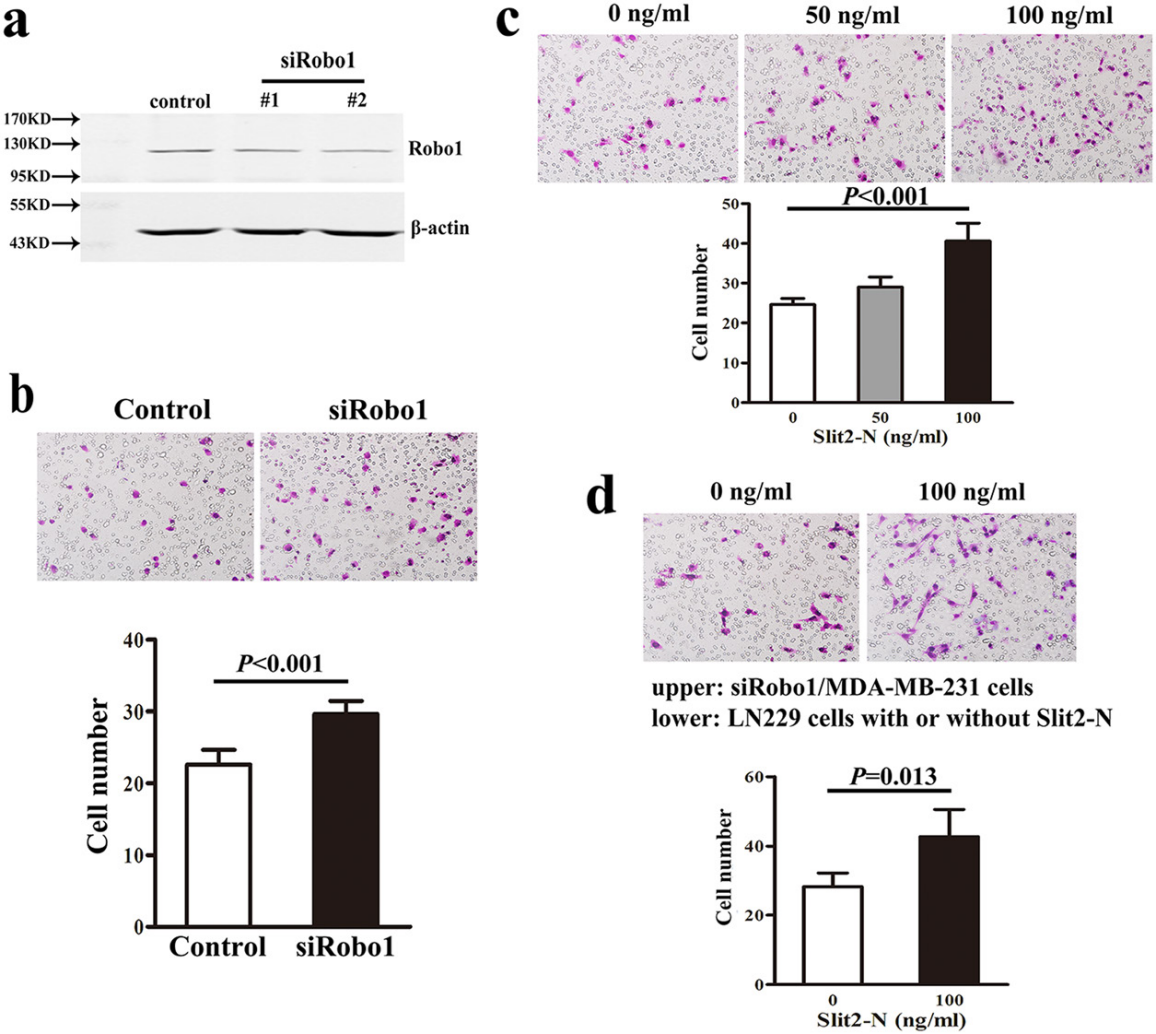
Function of Slit2/Robo1 in breast cancer cells was confirmed by migration assays *in vitro*. (**a**) Robo1 was knocked down by small RNA interference technology in MDA-MB-231 cells and 2 different RNA interference sequences were applied (#1 and #2). Robo1 expression was detected by Western Blot. (**b**) Photographs of control and siRobo1/MDA-MB-231 cells in migration assay (200×). The number of migrated cells was quantified by counting stained cells in random fields of the membrane (Student’s t test, *P* < 0.001). (**c**) Photographs of MDA-MB-231 cells in migration assay (200×). Recombinant human Slit2-N peptide (corresponding to the N-terminal portion of the full length Slit2 precursor) was used as a chemoattractant (50 ng/ml, 100 ng/ml) and the number of migrated cells was quantified (one-way AVOVA, *P* < 0.001). (**d**) Photographs of siRobo1/MDA-MB-231 cells in migration assay (200×). Co-culture of siRobo1/MDA-MB-231 cells (upper chamber) with brain derived cells (glioblastoma cell line LN229 in lower chamber with or without Slit2-N) were performed. The number of migrated cells was quantified (Student’s t-test, *P* = 0.013). All experiments were performed three times independently.

In order to confirm the role of Slit2/Robo1 in breast cancer, we performed the migration assays *in vitro*. First, we knocked down the expression of Robo1 in the MDA-MB-231 cells. 2 different RNA interference sequences were applied and we found #2 was more effective by Western Blot detection (Fig. 4a). Then we screened siRobo1/MDA-MB-231 cells by using #2 sequences and performed migration assay. The result showed cells migration was increased after down regulation of Robo1 (Student’s t test, *P* < 0.001, Fig. 4b). In the following, we applied recombinant human Slit2-N peptide (corresponding to the N-terminal portion of the full length Slit2 precursor) as a chemoattractant, and we found more MDA-MB-231 cells migrated into the lower chamber in the Slit2-N group (50 ng/ml or 100 ng/ml) than the control (One-way ANOVA, *P* < 0.001, Fig. 4c). Furthermore, co-culture of siRobo1/MDA-MB-231 cells (upper chamber) with brain derived cells (glioblastoma cell line LN229, lower chamber) were performed in the transwell system, we found LN229 cells with 100 ng/ml of Slit2-N in the lower chamber promoted the migration of siRobo1 cells compared with the Slit2-N absent group (Student’s t test, *P* = 0.013, Fig. 4d).

## Discussion

It is well established that Slit2/Robo1 axis plays an important role in breast development and morphology, loss of Slit2 or Robo1 resulted in a precocious branching phenotype characterized by an excess of disorganized basal myoepithelial cells^12^. Slit2 promoter hypermethylation in tissue and serum samples from breast cancer patients was a possible marker for early detection^13^. In this present study, we provided new evidence that Slit2/Robo1 axis is a suppressor of breast cancer progression. Moreover, low expression of Slit2 or Robo1 in breast cancer patients correlated with poor OS and DFS, suggesting Slit2/Robo1 axis can serve as a prognostic biomarker of breast cancer. Notably, our clinical data showed, for the first time, that low expression of Slit2 or Robo1 positively correlated with brain-specific metastasis of breast cancer patients, but not liver, bone or lung metastasis.

In retrospective studies, researchers found that low expression of Slit2 in breast cancer and pancreatic ductal adenocarcinoma (PDAC) patients were both associated with higher incidence and a higher extent of lymph node metastasis^14^,^15^. Furthermore, the expression of Robo1 in metastasized lymph nodes was significantly lower than in the primary tumors in intrahepatic cholangiocarcinoma^16^. All these results indicated that tumor cells with low Slit2/Robo1 expression potentially had metastatic intention.

Slit2 is a bifunctional guidance cue and it acts as not only a chemorepellent but also a chemoattractant^17^. Slit2 was regarded as a chemorepulsive factor to control migration of growth cones with high level of Robo1 during central nervous system development^18^. Slit2 secreted by midline glia could prevent axons with high level of Robo1 to cross the midline, while growth cones expressing low level of Robo1 were allowed to cross^18^,^19^. Schmid *et al.* demonstrated Slit2 could exert function as a chemokine to promote breast cancer cells migration^20^, which was consistent with our result of Fig. 4c. According to our results of Fig. 4d, we showed that LN229 cells with high concentration of Slit2-N in the lower chamber promoted the migration of siRobo1/MDA-MB-231 cells compared with the Slit2-N absent group. In addition, it was known that expression of Slit2 in both bone marrow and liver were much lower than brain^21^,^22^, therefore we hypothesized that high level of Slit2 in brain serving as a chemokine to attract breast cancer cells expressing low level of Robo1 was the one of the possibilities contributing to brain specific metastasis.

It was reported that inhibition of Slit2/Robo1 signaling could promote progression of breast cancer via activating PI3K/Akt/β-catenin pathway and accelerated translocation of β-catenin into nucleus *in vitro* and *in vivo*^14^. Studies indicated that down-regulation Slit2/Robo1 signaling in breast cancer cells led to an increased expression of MMP-9, which is one of target proteins of β-catenin^23^. MMP-9, as a member of MMP family, was reported to have higher expression in brain-selective breast cancer cells than that of parental and bone-selective cells^24^. Therefore, we assumed that Slit2/Robo1 axis has effects on breast cancer brain metastasis probably by modulation of PI3K/Akt/β-catenin/MMP-9 signaling.

Taken together, our findings indicated that the expression of Slit2/Robo1 axis possibly be regarded as a significant clinical parameter for predicting intention of brain metastasis in breast cancer patients and probably contributes to providing a new therapeutic target for patients with brain metastasis.

## Materials and Methods

### Patient selection and clinical information

Paraffin-embedded specimens from 118 breast cancer patients with invasive ductal carcinoma (IDC), diagnosed between 2003 and 2004, together with 44 cases of breast ductal carcinoma *in situ* (DCIS) and 34 cases of benign breast lesions were reviewed and selected from the archives of the Department of Breast Cancer Pathology and Research Laboratory, Tianjin Medical University Cancer Institute & Hospital (Tianjin, China). This study was reviewed and approved by the Ethic Committee of Tianjin Medical University Cancer Institute & Hospital. All experiments were performed in accordance with relevant guidelines and regulations of Ethic Committee of Tianjin Medical University Cancer Institute & Hospital. All the patients signed an informed consent for participation of the study and the use of their biological tissues. The histopathology was reviewed and the diagnosis in each case was confirmed independently by three pathologists according to World Health Organization (WHO) criteria.

The median age of the IDC patients was 50 years old (range, 28–79). None of them had received neo-adjuvant chemotherapy or preoperative radiation therapy. The patients were followed up for 2–124 months during which 5 (4.23%) patients suffered local or regional tumor recurrence, 23 (19.3%) developed distant metastasis, and 13 (10.9%) patients died of tumors.

Among the 23 patients who developed distant metastasis during the follow-up period, 18 patients developed bone metastasis, 9 developed lung metastasis, 10 developed liver metastasis and 8 developed brain metastasis. It is worth noting that multiple organic metastases were noted in 13 patients.

To further investigate the possible mechanism of brain metastasis of breast cancer, an enlarged cohort of 33 patients with brain metastasis, mean aged 48.58 (range, 28–70) were selected. Notably, 14 matched pairs of primary and brain metastasis tumors were collected.

### Immunohistochemical staining

Formalin-fixed, paraffin-embedded serial tissue sections from each case were selected. Slit2 and Robo1 were stained by S-P method. In brief, 5 μm tissue sections were subsequently dewaxed and rehydrated using xylene and graded alcohol washes. Antigen retrieval was performed at 121 °C for 2 min 15 seconds, using citrate buffer, pH 6.0. After serial blocking with hydrogen peroxide and normal horse serum, the sections were incubated with primary monoclonal antibody against Slit2 (goat polyclonal 1:50, Santa Cruz, USA.) or Robo1 (goat polyclonal 1:50, Santa Cruz, USA.) for 16 h at 4 °C. The sections were then sequentially incubated with biotinylated rabbit anti-goat immunoglobulin and peroxidase-conjugated streptavidin. The enzyme substrate was 3,3′-diaminobenzidinetetra-hydrochloride (DAB). Immunochemistry staining without primary antibody was regarded as negative control.

### Evaluation of staining

The tissue sections stained immunohistochemically for Slit2 and Robo1 were reviewed and scored separately by two pathologists blinded to the clinical parameters. The third pathologist arbitrated any disagreements. A consensus judgment was adopted for the intensity score of the tumors based on the strength of Robo1 and Slit2 expression: 0 (−) no staining; 1 (+) definite but weak staining; 2 (++) moderate staining; 3 (+++) strong staining. Percentage of the positive staining is scored as follows: 0 (0–10%); 1 (11–49%); 2 (50–100%). An immunohistochemical score (IHC score) was obtained ranged from 0 to 6 by multiplying the intensity and the percentage score. Patients were categorized into groups according to IHC score of Slit2 or Robo1: low Slit2 expression (0–2), high Slit2 expression (3–6), low Robo1 expression (0–2), and high Robo1 expression (3–6).

### Cell Culture and Reagents

MDA-MB-231 breast cancer cells and LN229 glioblastoma cells were cultured in Dulbecco’s modified Eagle medium (DMEM), supplemented with 10% fetal bovine serum (FBS), 100 units/ml penicillin and 100 μg/ml streptomycin, in a 5% CO_2_ incubator at 37 °C. Cells had been tested and authenticated by DNA (STR) profiling, work performed by Beijing Microread Genetics Co., Ltd. (Beijing, China). Antibodies used in Western Blot for β-actin (sc-47778) and Robo1 (sc-25672) were acquired from Santa Cruz Biotechnology (Santa Cruz, CA). Fluorescent secondary antibody used for Western Blots include anti-rabbit antibody (926-32211, Odyssey LiCor, IR800) and anti-mouse antibody (926-32210, Odyssey LiCor, IR700). Recombinant human Slit2-N (#150-11, a 1093 amino acid glycoprotein corresponding to the N-terminal portion of the full length Slit2 precursor) was purchased from PeproTech (Rocky Hill, NJ).

### Lentvirus Production and Infection

Robo1-specific siRNA sequences were designed according to the previous report^25^. 2 different RNA interference sequences were applied (#1 and #2). The sequences were following: (5′- CCGGAAGGCATATTTGGAAGTTACACTCGAGTGTAACTTCCAAATATGCCTTTTTTT-3′) (#1); (5′- CCGGGCCCACCATTTCATGGAAGAACTCGAGTTCTTCCATGAAATGGTGGGCTTTTT -3′) (#2); scrambled sequence (5′-CCGGGTTCCCGAACGTGTCACGTCTCGAGACGTGACACGTTCGGAGAACTTTTT -3′). Scrambled sequence was regarded as control. They were synthesized and cloned into pLKO.1 pure vector. Lentiviruses were produced by cotransfection of lentiviral vector and packing plasmids ΔR and pVSVg with calcium chloride solution and HBS (50 mM HEPES, 280 mM NaCl, 1.5 mM Na_2_HPO_4_, pH 7.0) into HEK-293T cells. Supernatant was collected and the virus was used to infect cultured MDA-MB-231 cells. Expression of Robo1 was verified by Western Blot analysis.

### Western Blot

Cells were lysed in 1 × SDS lysis buffer (Tris-HCl, pH 6.8, 62.5 mM, 2% SDS, 10% glycerol) followed by centrifugation. Equal amounts of cell lysates were loaded and separated by SDS-PAGE, and proteins were transferred onto nitrocellulose membranes and incubated overnight at 4 °C with the primary antibody. Membranes were next treated with secondary antibodies for 1 hour and then analyzed by using the LiCor Odyssey infrared imaging.

### Migration assay

Migration assays were performed using 24-well transwell migration chambers (Corning, Corning, New York, USA) with polyethylene membranes (8 μm pore size). The upper chambers were seeded with 2.5 × 10^4^ cells/well in 200 μl of serum-free DMEM supplemented with 0.1% BSA. The cells were allowed to migrate for 10 h at 37 °C. In co-culture migration assays, 3 × 10^4^ LN229 cells/well were seeded in lower chamber 12 h before experiments. Afterward, cells at the upper layer of the membrane were scraped and cells at the lower layer were stained with Giemsa solution and photographed under a microscope. The number of cells was quantified in randomly selected fields. Three independent experiments were performed.

### Statistical Methods

Differences in categorical variables were evaluated with the χ^2^ test. The relation between Slit2 or Robo1 expression and distance metastasis was evaluated using Spearman rank correlation analysis. Mann-Whitney U test was used for analyzing the expression differences of Slit2 and Robo1 in patients with or without brain metastasis. To compare IHC score of Slit2 and Robo1 in paired primary and brain metastatic tumors, Wilcoxon signed ranks test was performed. Disease-free survival was defined as the time from surgery to recurrence or cancer-specific death, whichever occurred first. Overall survival (OS) was calculated from histologic diagnosis to the date of last contact or death from breast carcinoma. Survival analysis was performed according to the Kaplan-Meier method, univariate and multivariate Cox proportional hazard regression analysis. Interval from diagnosis of breast cancer to development of brain metastasis and survival after brain metastasis was estimated using Kaplan-Meier method, and they were compared using Mann-Whitney U test.

For *in vitro* experiments, all data was expressed as mean ± standard deviation (SD). The independent-samples *t* test was used to evaluate the statistical significance between 2 groups and One-way ANOVA analysis was used to evaluate the statistical significance of the difference between more than 2 groups. SPSS13.0 statistical package were used throughout. All statistical tests were 2-tailed and *P* < 0.05 was regarded as significant.

## Additional Information

**How to cite this article**: Qin, F. *et al.* Low Expression of Slit2 and Robo1 is Associated with Poor Prognosis and Brain-specific Metastasis of Breast Cancer Patients. *Sci. Rep.* **5**, 14430; doi: 10.1038/srep14430 (2015).

## Supplementary Material

Supplementary Information

## Figures and Tables

**Table 1:** Slit2 and Robo1 expression in different breast tissue specimens.

**Pathological type**	**n**	**Slit2 score, n (%)**		**χ^2^**	***P***	**Robo1 score, n (%)**		**χ^2^**	***P***
		**0–2**	**3–6**			**0–2**	**3–6**		
Benign lesion	34	5 (14.7)	29 (85.3)	7.724	0.024	7 (20.6)	27 (79.4)	7.056	0.029
DCIS	44	16 (36.4)	28 (63.6)			19 (43.2)	25 (56.8)		
IDC	118	47 (39.8)	71 (60.2)			54 (45.8)	64 (54.2)		

DCIS: ductal carcinoma *in situ*; IDC: invasive ductal carcinoma.

Slit2: DCIS vs. Benign lesion: *P* = 0.032, χ^2^ = 4.573; IDC vs. Benign lesion: *P* = 0.007, χ^2^ = 7.403; DCIS vs. IDC: *P* = 0.687, χ^2^ = 0.162.

Robo1: DCIS vs. Benign lesion: *P* = 0.036, χ^2^ = 4.406; IDC vs. Benign lesion: *P* = 0.008, χ^2^ = 6.962; DCIS vs. IDC: *P* = 0.769, χ^2^ = 0.086.

**Table 2:** Univariate and Multivariate proportional hazards analysis of overall survival (OS) in 118 IDC patients.

	**Univariate analysis**		**Multivariate analysis for Slit2**		**Multivariate analysis for Robo1**	
	**HR (95% CI)**	***P***	**HR (95% CI)**	***P***	**HR (95% CI)**	***P***
Tumor size	1.243 (0.409**–**3.779)	0.701	0.379 (0.105**–**1.375)	0.379	0.466 (0.119**–**1.824)	0.273
cTNM	4.382 (1.732**–**11.091)	0.002	3.888 (1.129**–**13.390)	0.031	3.142 (0.897**–**11.007)	0.073
Lymph node metastasis	2.919 (1.718–4.960)	0.000	2.530 (1.400**–**4.569)	0.002	2.414 (1.333**–**4.373)	0.004
Pathological grade	4.095 (1.528**–**10.972)	0.005	9.021 (2.323**–**35.030)	0.001	7.408 (1.965**–**27.925)	0.003
Slit2 expression	0.285 (0.088**–**0.925)	0.037	0.088 (0.016**–**0.502)	0.006		
Robo1 expression	0.227 (0.062**–**0.824)	0.024			0.142 (0.030**–**0.666)	0.013

HR: Hazard ratio; CI: confidence interval.

**Table 3:** Relationship between Slit2 (or Robo1) expression and distance metastasis in 118 IDC patients.

**Distant metastases**	**Slit2 score, n (%)**		***r*_s_**	***P***	**Robo1 score, n (%)**		***r*_s_**	***P***
	**0–2**	**3–6**			**0–2**	**3–6**		
Brain metastasis			−0.194	0.036			−0.226	0.014
No	41 (37.3)	69 (62.7)			47 (42.7)	63 (57.3)		
Yes	6 (75.0)	2 (25.0)			7 (87.5)	1 (12.5)		
Bone metastasis			−0.136	0.141			−0.131	0.158
No	37 (37.0)	63 (63.0)			43 (43.0)	57 (57.0)		
Yes	10 (55.6)	8 (44.4)			11 (61.1)	7 (38.9)		
Lung metastasis			−0.027	0.771			−0.056	0.544
No	43 (39.4)	66 (60.6)			49 (45.0)	60 (55.0)		
Yes	4 (44.4)	5 (55.6)			5 (55.6)	4 (44.4)		
Liver metastasis			−0.063	0.496			−0.148	0.110
No	42 (38.9)	66 (61.1)			47 (43.5)	61 (56.5)		
Yes	5 (50.0)	5 (50.0)			7 (70.0)	3 (30.0)		

**Table 4:** Slit2 or Robo1 expression in patients with or without brain metastasis.

**Brain metastases**	**n**	**Slit2 score, n (%)**		**χ^2^**	***P***	**Robo1 score, n (%)**		**χ^2^**	***P***
		**0–2**	**3–6**			**0–2**	**3–6**		
Yes	33	23 (69.7)	10 (30.3)	10.794	0.001	25 (75.8)	8 (24.2)	11.078	0.001
No	110	41 (37.3)	69 (62.7)			47 (42.7)	63 (57.3)		
