# Supplementary material for: Low Expression of Slit2 and Robo1 is Associated with Poor Prognosis and Brain-specific Metastasis of Breast Cancer Patients
Source: Sci Rep. 2015 Sep 24;5:14430. doi: 10.1038/srep14430 (PMC4585856; doi:10.1038/srep14430)
Supplement: Supplementary Information [file srep14430-s1.pdf]

# Low expression of Slit2 and Robo1 is Associated with poor prognosis and brain-specific metastasis of breast cancer patients

Fengxia Qin[1], Huikun Zhang[1], Li Ma[2], Xiaoli Liu[3], Kun Dai[1], Wenliang Li[2], Feng Gu[1]*, Li Fu[1]*, Yongjie Ma[3]*

**Supplementary FigureS1**

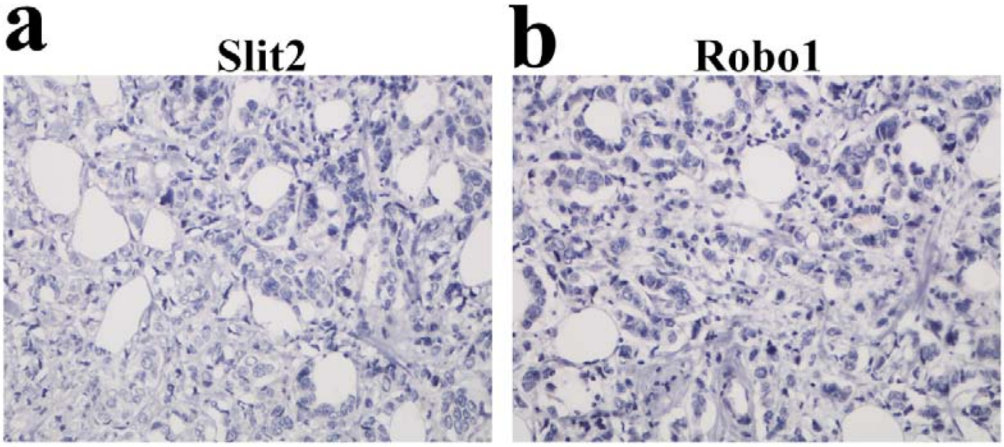


**Supplementary Figure Legend**

**Figure S1:** Negative controls of immunohistochemical analysis (200×). (a) Image of the negative control for Slit2 (without primary antibody). (b) Image of the negative control for Robo1 (without primary antibody).

**Supplementary Table S1. Correlation between Slit2 and Robo1 expression in IDC**

| Slit2 score | Robo1 score, n (%) | | $r_s$ | *P* value |
|---|---|---|---|---|
| | 0-2 | 3-6 | | |
| 0-2 | 37 (78.7) | 10 (21.3) | 0.538 | 0.000 |
| 3-6 | 17 (23.9) | 54 (76.1) | | |

**Supplementary Table S2. Clinicopathologic characteristics of patients with or without brain metastasis**

| Variables | Brain metastasis | | $\chi^2$ | *P* value |
|---|---|---|---|---|
| | Negative | Positive | | |
| Age, y | | | 0.841 | 0.359 |
| ＜50 | 50 (73.5) | 18 (26.5) | | |
| ⩾50 | 60 (80.0) | 15 (20.0) | | |
| Tumor size (cm) | | | 1.063 | 0.588 |
| ⩽2 | 21 (72.4) | 8 (27.6) | | |
| 2-5 | 80 (79.2) | 21 (20.8) | | |
| ＞5 | 9 (69.2) | 4 (30.8) | | |
| Pathological stage | | | 0.857 | 0.651 |
| I | 17 (85.0) | 3 (15.0) | | |
| II | 74 (75.5) | 24 (24.5) | | |
| III- IV | 19 (76.0) | 6 (24.0) | | |
| Pathological grade | | | 2.336 | 0.311 |
| I | 17 (77.3) | 5 (22.7) | | |
| II | 73 (80.2) | 18 (19.8) | | |
| III | 20 (66.7) | 10 (33.3) | | |
| Lymph node metastases | | | 0.896 | 0.344 |
| Negative | 53 (73.6) | 19 (26.4) | | |
| Positive | 57 (80.3) | 14 (19.7) | | |
